# Enhanced sunlight driven photocatalytic activity of In_2_S_3_ nanosheets functionalized MoS_2_ nanoflowers heterostructures

**DOI:** 10.1038/s41598-021-94966-z

**Published:** 2021-07-28

**Authors:** Jaspal Singh, R. K. Soni

**Affiliations:** Laser Spectroscopy Lab, Department of Physics, Indian Institute of Technology Delhi, Hauz Khas, New Delhi, 110016 India

**Keywords:** Energy science and technology, Nanoscience and technology

## Abstract

Visible light-sensitive 2D-layered based photocatalytic systems have been proven one of the effective recent trends. We report the preparation of a 2D-layered based In_2_S_3_–MoS_2_ nanohybrid system through a facile hydrothermal method, capable of efficiently degrading of organic contaminants with remarkable efficiency. Transmission electron microscopy (TEM) results inferred the attachment of 2D-layered In_2_S_3_ sheets with the MoS_2_ nanoflakes. Field emission SEM studies with chemical mapping confirm the uniform distribution of Mo, In, and S atoms in the heterostructure, affirming sample uniformity. X-ray diffraction, X-ray photoelectron spectroscopy, and Raman spectroscopy results confirm the appearance of 2H-MoS_2_ and β-In_2_S_3_ in the grown heterostructures. UV-DRS results reveal a significant improvement in the optical absorbance and significant bandgap narrowing (0.43 eV) in In_2_S_3_–MoS_2_ nanohybrid compared to pristine In_2_S_3_ nanosheets in the visible region. The effective bandgap narrowing facilitates the charge transfer between MoS_2_ and In_2_S_3_ and remarkably improves the synergistic effect. Effective bandgap engineering and improved optical absorption of In_2_S_3_–MoS_2_ nanohybrids are favorable for enhancing their charge separation and photocatalytic ability. The photocatalytic decomposition efficiency of the pristine In_2_S_3_ nanosheets and In_2_S_3_–MoS_2_ nanohybrids sample is determined by the decomposing of methylene blue and oxytetracycline molecules under natural sunlight. The optimized In_2_S_3_–MoS_2_ nanohybrids can decompose 97.67% of MB and 76.3% of OTC-HCl molecules solution in 8 min and 40 min of exposure of sunlight respectively. 2D-layered In_2_S_3_-MoS_2_ nanohybrids reveal the tremendous remediation performance towards chemical contaminations and pharmaceutical waste, which indicates their applicability in industrial and practical applications.

## Introduction

Visible light-sensitive photocatalyst has been extensively studied due to their tremendous performance for energy production and environmental remediation applications^1–5^. Visible light-sensitive photocatalyst indicated the capability and usage of engineered nanostructures into the light-harvesting capability from natural sunlight. For more advanced and practical applications, photocatalyst material should be highly sensitive towards visible light, which can strongly absorb the visible spectrum from sunlight. Semiconductor-based photocatalysts have been proven to be one of the best ways to decomposed organic waste using light exposure after the outstanding discovery of water splitting by Fujishima in 1972^6–15^. The efficiency of semiconductor-based photocatalyst, however, is limited by insufficient light utilization and poor effective surface area. To overcome these issues, two-dimensional (2D) layered structured transition metal chalcogenides (TMDC) based photocatalysts have been developed, which tremendously absorb the visible light and also provide sufficient effective surface area^16–18^. In addition, layered structured TMDC based photocatalyst exhibits outstanding chemical, optical and electronic properties which make them unique for environmental remediation and energy production applications^19,20^. Among various layered structures Indium sulfide (In_2_S_3_) has received significant attention recently in the field of visible light photocatalyst because of its low toxicity, high photo-stability, and narrow bandgap (2.0–2.3 eV)^21,22^. On the other hand, the photocatalytic nature of In_2_S_3_ is found to be low due to the high recombination rate and poor mass transfer^23^. Several parameters such as modulation in morphology, a surface area strongly influenced the photocatalytic activity of the In_2_S_3_ nanostructures by efficient charge separation, the improved lifetime of charge carriers, and high rate of mass transfer^23,24^. Apart from this, various efforts, such as phase optimization^25,26^, heterostructures creation^27,28^, and noble metal nanoparticle functionalization^29,30^, have been devoted to improving the photodegradation capability of In_2_S_3_ nanostructures. 2D-TMDCs based heterostructures-based photocatalyst is one of the advanced and effective strategies to control the recombination rate and improve the photodegradation ability through synergistic effect between two layered nanostructures^31^. Nanostructured MoS_2_ (bandgap ~ 1.9 eV) is extensively explored and employed for several applications such as sensing^32^, energy production^33^, optoelectronic devices^34^, and antibacterial activity^35^. Due to its fascinating optical, electronic, and chemical properties. MoS_2_ modified In_2_S_3_ based heterostructures can effectively enhance the lifetime of photoinduced charge carries through synergistic effect among them^36^. In_2_S_3_ nanosheets combined with MoS_2_ nanoflowers is expected to increase the photoinduced catalytic activity^37–39^ due to sharing of the similar 2D layered structures by MoS_2_ and In_2_S_3,_ which inferred the creation of high-quality, intimate heterojunction, and exhibits preferential band-gap alignments that help to generate the unsaturated radicals to enhance the rate of photocatalytic reactions. It has been found that the existence of MoS_2_ can initiate the formation of superoxide radicals which is beneficial for the decomposition of organic molecules under light exposure^40,41^. Moreover, MoS_2_ presence in In_2_S_3_ can further enhance the active sites, which can effectively interact with the pollutant molecules. Li et al.^23^ fabricated hierarchical In_2_S_3_/MoS_2_ nanosheets using the exfoliation method with the hydrothermal method. The prepared In_2_S_3_/MoS_2_ nanohybrids were embraced for photocatalytic application through the Aza-Henry reaction. They have demonstrated that In_2_S_3_/MoS_2_ nanohybrids attained superior photocatalytic activity compared to pure MoS_2_ and In_2_S_3_. Sun et al.^31^ prepared MoS_2_/In_2_S_3_ flakes-based photoanode by using a one-pot synthesis process and applied it for the H_2_ production application. In their study, they have demonstrated the effective charge separation in MoS_2_/In_2_S_3_ flakes for a high H_2_ production rate.

2D-layered-based heterostructures are emerging as the new material for environmental remediation applications and are rarely reported in the literature. Removal of pharmaceutical and chemical waste has not been reported by using the 2D-layered In_2_S_3_-MoS_2_ nanohybrids-based photocatalyst. This study also highlights the contribution of improvement in the optical absorption and significant bandgap narrowing on the photodegradation nature of In_2_S_3_-MoS_2_ nanoheterostructures.

In this report, we highlight the synergistic effect due to the effective bandgap narrowing in 2D-layered-based heterostructures for pollutant removal. To employ the proposed strategy, 2D-layered-based In_2_S_3_–MoS_2_ nanoheterostructures were engineered using a hydrothermal method and determined their photodegradation capability to decompose the variety of organic pollutants molecules methylene blue and oxytetracycline under sunlight illumination. Chemical surface states, optical profile, and modulation in the morphologies are explored and assured in the hierarchical heterostructures of In_2_S_3_ and MoS_2_. In_2_S_3_–MoS_2_ nanoheterostructures exhibit a significantly superior photocatalytic nature as compared to pristine In_2_S_3_ nanostructures. We have tuned the heterojunction density by varying the amount of MoS_2_ over In_2_S_3_ for a superior photodegradation process which provides the insight understanding to design the efficient photocatalyst. Apart from this, In_2_S_3_–MoS_2_ nanoheterostructures revealed significant reusability and stability, which indicate their possible applications for other sunlight-driven processes.

## Experimental

### Materials

Indium chloride, Hexaammonium heptamolybdate tetrahydrate, and thiourea were purchased from Sigma-Aldrich, while oxytetracycline (OTC HCl) and methylene blue (MB) were obtained from SRL, and Merck, respectively. All chemical reagents were employed as purchased.

### Formation of β-In_2_S_3_-2H-MoS_2_ heterostructures photocatalyst

**Figure 1:**
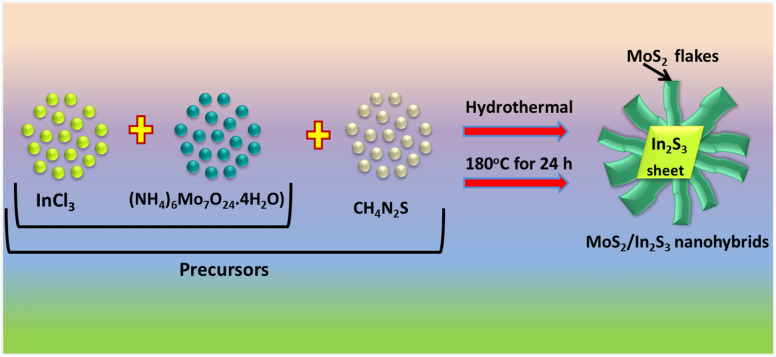
Depicts the fabrication process of In_2_S_3_–MoS_2_ nanohybrids.

In the reaction process, initially, 20 mL of Hexaammonium heptamolybdate tetrahydrate (0.08 mM) was dropwise added into the 20 mL thiourea (0.18 mM) under constant stirring. Similarly, another solution contained 20 mL of indium chloride (0.24 mM) was dropwise added into the 20 mL thiourea solution. In the next step, both solutions were mixed in a conical flask under vigorous stirring, after confirming the formation of a uniform mixture, it was transferred to a Teflon container of 100 mL. The pH of the obtained solution was set at 6 by using 0.5 M NaOH solution. In the next step, the Teflon-lined stainless steel autoclave contained sample was held at 180 °C for 18 h. The obtained sample was treated with ethanol and DI water and collected by centrifugation process, and finally, placed at 80 °C in the oven. The pristine In_2_S_3_ and In_2_S_3_–MoS_2_ samples with tuneable MoS_2_ density (0.16 mM and 0.24 mM) were generated by the above-mentioned process. For better understanding, pristine In_2_S_3_ and In_2_S_3_-MoS_2_ heterostructures (0.08 mM, 0.16 mM and 0.24 mM) hereafter reported as IP, IPM1, IPM2 and IPM3, respectively. The reaction process is depicted in Fig. 1.

### Characterization techniques and photocatalysis reaction

Surface morphologies of pristine In_2_S_3_ and MoS_2_ modified In_2_S_3_ samples were explored through scanning electron microscopy (ZEISS, EVO) combined with the elemental mapping facility, while transmission electron microscopy (JEOL-2100 F, Japan) was employed to determine the crystal structure of In_2_S_3_-MoS_2_ nanohybrids. Powder X-ray diffraction technique (Rigaku Ultima IV, Ri) was employed to investigate the diffraction patterns of In_2_S_3_–MoS_2_ nanohybrids. Optical studies of synthesized samples were explored by the UV-DRS (Shimadzu UV-2450) method, photoluminescence spectroscopy (RF-6000 Shimadzu), and Raman spectroscopy (Renishaw inVia). The chemical composition of In_2_S_3_–MoS_2_ nanohybrids was investigated through X-ray photoelectron spectroscopy (ESCA + Omicron Nano Technology). The photodegradation ability of MoS_2_ functionalized In_2_S_3_ and pure In_2_S_3_samples ware explored by the breakdown of 10 µM MB solution and oxytetracycline (OTC-HCl) molecules in sunlight exposure (800 W/m^2^). Modulations in the intensity of the targeted pollutant molecules solutions with different photocatalyst samples for the same exposure time intervals (2, 4, 6, and 8 min) were measured using UV–Visible absorption spectroscopy (Perkin Elmer). In the photocatalytic reaction, the used amount of each photocatalyst sample is 2.5 mg/L. In order to explore the active species in the photodegradation reaction charge trapping studies were performed. To trap the superoxide radicals, electrons, hydroxyl radicals, and holes four scavengers namely benzoquinone (BQ), copper nitrate (CN), formic acid (FA), and Isopropanol alcohol (IPA) were employed, respectively.

## Results and discussion

**Figure 2:**
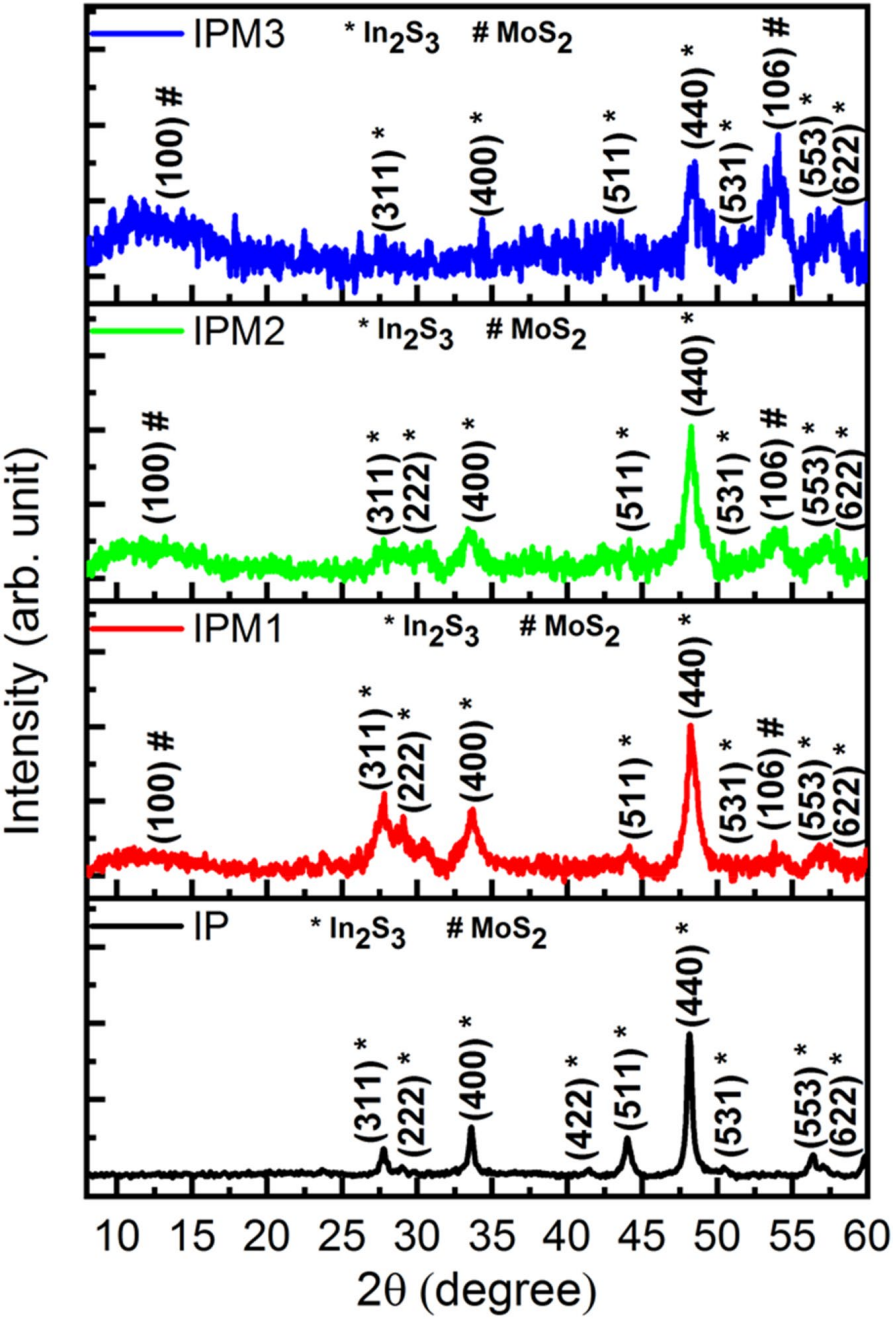
X-ray diffraction reflections of In_2_S_3_ nanosheets and In_2_S_3_–MoS_2_ nanohybrids.

Figure 2 illustrates the X-ray diffraction patterns of pristine In_2_S_3_ and MoS_2_ modified In_2_S_3_. XRD for sample IP indicates nine reflections (311), (222), (400), (422), (511), (440), (531), (553), and (622), which assured the presence of β-phase of In_2_S_3_ (JCPDS-500814). XRD spectrum for sample IPM1 shows eight diffraction patterns (311), (222), (400), (511), (440), (531), (533), and (622) corresponds to In_2_S_3_ while two peaks with reflection (100) and (106) assures the existence of 2H phase of MoS_2_ (JCPDS-371492). XRD spectrum for sample IPM2 indicates similar diffraction peaks compared to the XRD curve of sample IPM1. Interestingly, the peak intensity of (100) and (106) peaks are found enhanced in sample IPM2 compared to sample IPM1 which inferred the high concentration of MoS_2_ in In_2_S_3_–MoS_2_ nanohybrids. For sample IPM3, nine diffraction peaks (100), (311), (400), (511), (440), (531), (106), (553) and (622) can be appeared. Among them, seven peaks (311), (400), (511), (440), (531), (553), and (622) inferred the formation of In_2_S_3_ while (100) and (106) indicate the presence of MoS_2_. XRD results assure the formation of In_2_S_3_–MoS_2_ nanohybrids in samples IPM1, IPM2 and, IPM3.

**Figure 3:**
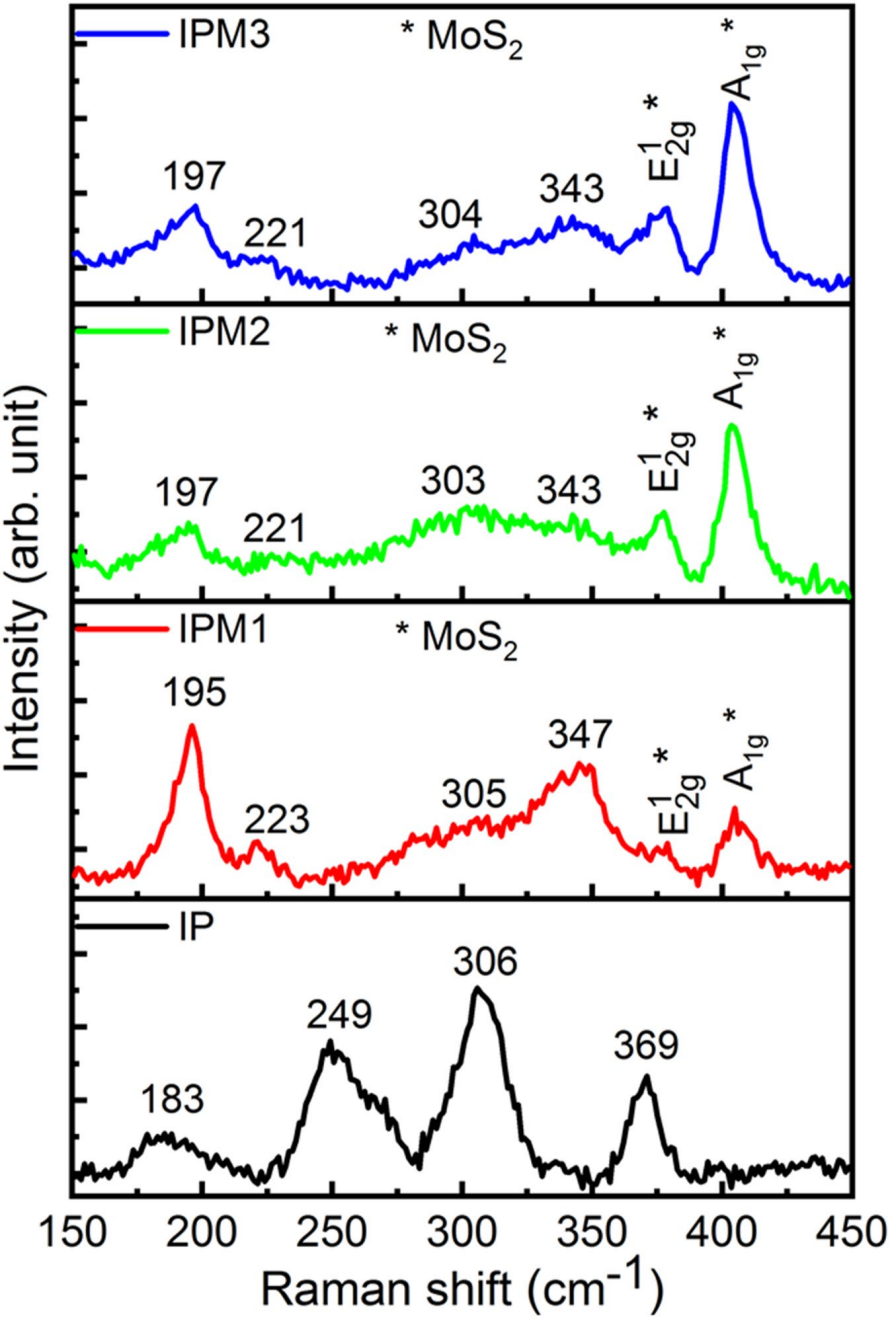
Raman results of In_2_S_3_ nanosheets and In_2_S_3_–MoS_2_ nanohybrids.

Raman spectra of sample IP, IPM1, IPM2, and IPM3 are presented in Fig. 3. Raman spectrum for sample IP indicating the four distinct peaks at 183 cm^−1^, 249 cm^−1^, 306 cm^−1^ and 369 cm^−1^ which are attributed to the fingerprint vibrations of β-phase of In_2_S_3_^42^. Raman result for sample IPM1 indicates the slight shift in the Raman peaks corresponds to the β-phase of In_2_S_3_. The observed peaks are 195 cm^−1^, 223 cm^−1^, 305 cm^−1^ and 347 cm^−1^ implies the formation of MoS_2_–In_2_S_3_ nanohybrids^23,38^. Apart from this, two distinct peaks at 379 cm^−1^ and 405 cm^−1^ arise in the Raman curve of sample IPM1, further confirmed the existence of MoS_2_. Raman spectrum for sample IPM2, the peaks correspond to the β-In_2_S_3_ and 2H-MoS_2_ are found slightly shifted compared to Raman spectrum of sample IPM1. The observed peaks for sample IPM2 are 197 cm^−1^, 221 cm^−1^, 303 cm^−1^, 343 cm^−1^, 378 cm^−1^ and 403 cm^−1^. Interestingly. It can be seen that the intensity of peaks at 378 cm^−1^ and 403 cm^−1^ is higher as than sample IPM1, which suggested the high-density MoS_2_ in sample IPM2 as compared to sample IPM1. For sample IPM3, the Raman studies reveals the six peak at 197 cm^−1^, 221 cm^−1^, 304 cm^−1^, 343 cm^−1^, 378 cm^−1^ and 405 cm^−1^. The intensity of two peaks for MoS_2_ at 378 cm^−1^ and 405 cm^−1^ in sample IPM3 is higher than that of sample IPM1 and IPM2. The significant shift in the Raman peaks of sample IPM1, IPM2, and IPM3 compared to sample IP affirms the attachment of MoS_2_ with In_2_S_3_^38^. The shift in the Raman spectra of MoS_2_ modified In_2_S_3_ can be ascribed due to the lattice vibrations enable through the variation in the bond force constant of Mo-S and In-S bond. Modulation in bond force constant generated due to the modification of Mo^4+^ atom in In_2_S_3_^23,38,43^.

**Figure 4:**
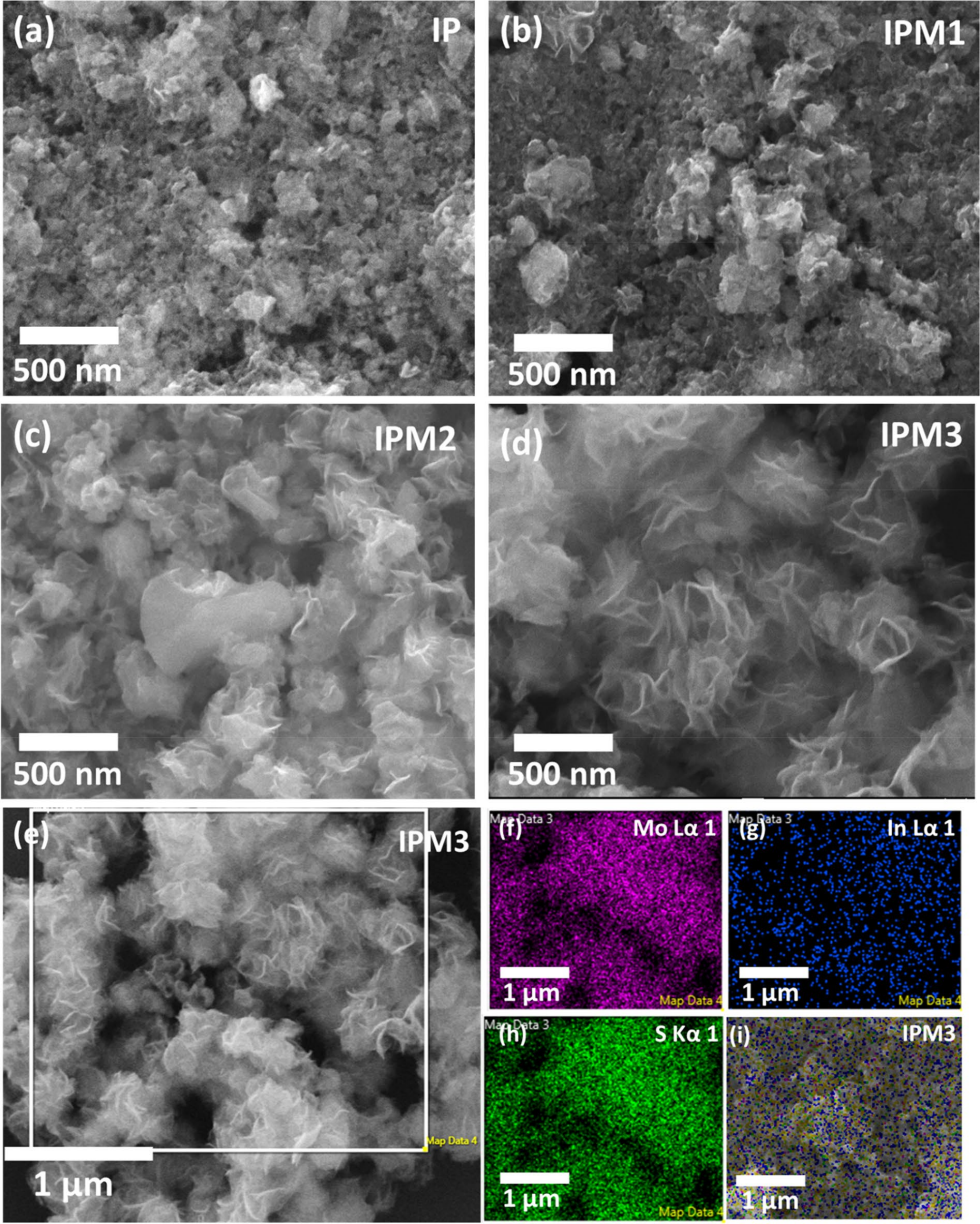
(**a**–**d**) FESEM images of sample IP, IPM1, IPM2, and IPM3 indicating the modulations in their morphology, (**e**) FESEM image of sample IPM3 revealing the formation of In_2_S_3_ nanosheet decorated MoS_2_ nanoflowers, (**f**–**h**) Chemical mapping of sample IPM3 assuring the independent existence of In, Mo, and S atoms in sample IPM3, (**i**) Mapped image of sample IPM3 assuring the formation of In_2_S_3_–MoS_2_.

**Figure 5:**
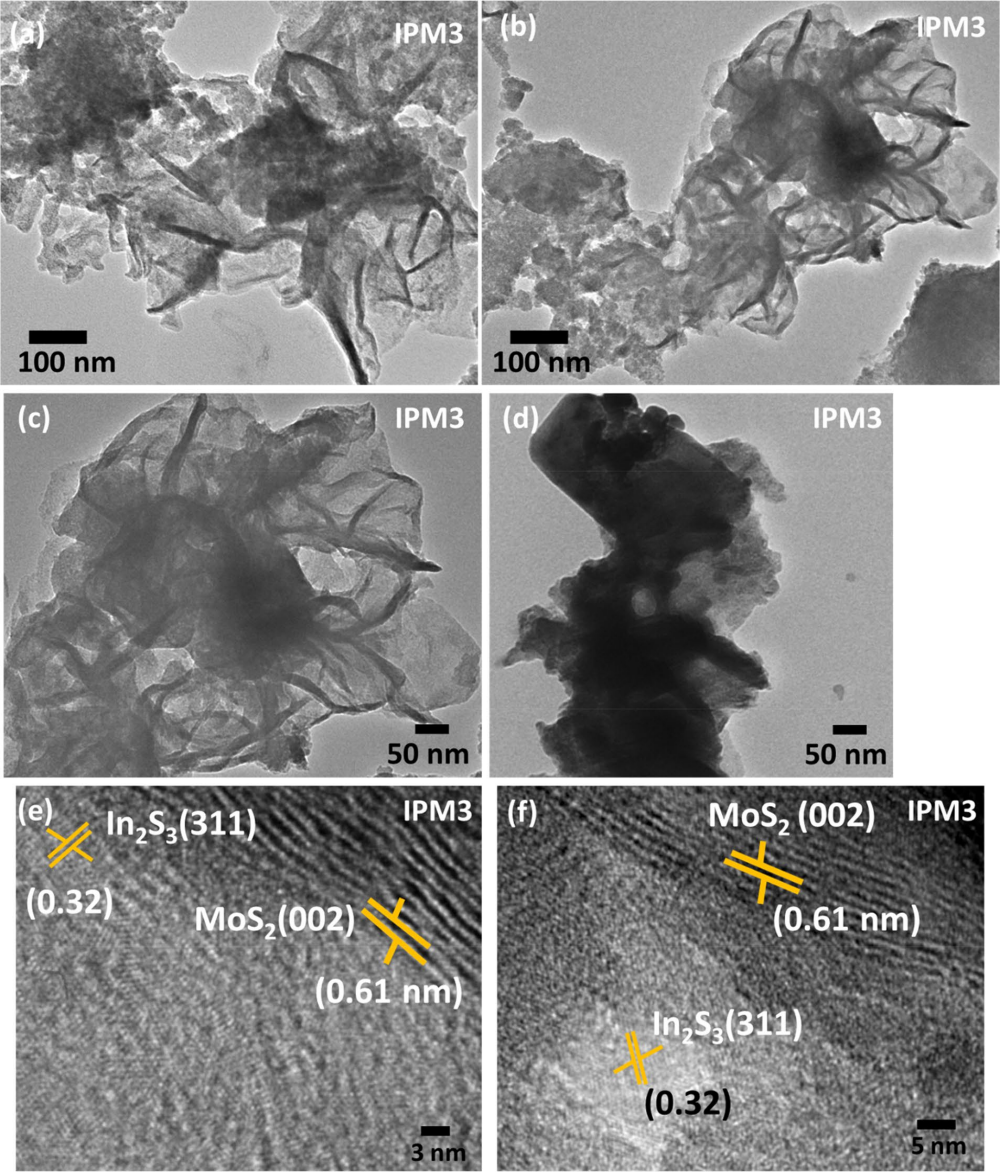
(**a**–**d**) TEM image of sample IPM3 revealing the formation of In_2_S_3_ nanosheet decorated MoS_2_ nanoflowers, (**e**, **f**) High-resolution TEM images indicating fringes distance for MoS_2_ and In_2_S_3._

Figure 4a–d depicts the modulations in the morphologies of sample IP, IPM1, IPM2, and IPM3. For sample IP, 2D- nanosheets can appear with an average width of 162 nm (Fig. 4a).FESEM result for sample IPM1 illustrates the functionalization of 2D-layered structured aggregates on sheet-like nanostructures Fig. 4b. With the increment in the concentration of MoS_2_, a more distinct flower-like morphology can be seen in the FESEM image of sample IPM2. Apart from this, a 2D-layered sheet can also be seen attached to the surface of flowers-like morphology (Fig. 4c). To further increase the Mo^+4^ ions concentration in In_2_S_3_, the 2D sheet mediated flowers-like structures attached with the sheet-like nanostructures can be observed for sample IPM3 (Fig. 4d). To confirm the formation of MoS_2_–In_2_S_3_ nanohybrids, elemental mapping for sample IPM3 was carried out and illustrated in Fig. 4e–i. The broader view of surface morphologies of sample IPM3 has been presented in Fig. 4e, while elemental mapping of the specific atom and corresponding nanohybrid is depicted in Fig. 4f–i. Elemental mapping studies affirm the uniform spreading of Mo, In, and S atoms in sample IPM3. Surface morphology results confirm the formation of MoS_2_–In_2_S_3_ nanohybrids. The EDX spectrum for sample IPM3 is presented in ((Fig. 1) supporting information). To explore the crystal structures and the attachment of In_2_S_3_ and MoS_2_, TEM and HRTEM studies were employed. Figure 5a–d shows the TEM images for sample IPM3 which inferred the presence of MoS_2_ nanoflakes were attached with the In_2_S_3_ nanosheets. Figure 5c,d reveals the heterojunction creation among the MoS_2_ nanoflakes and In_2_S_3_ sheets. TEM studies affirm that the assembly of MoS_2_ nanosheets formed the flowers-like nanostructures on the surface of In_2_S_3_ sheets. To further assured the formation of In_2_S_3_–MoS_2_ nanoheterojunction, high-resolution TEM studies were performed and depicted in Fig. 5e,f. The evaluated distance among the inter-planer lattice fringes is 0.61 nm and 0.32 nm which implies the existence of MoS_2_ (002) and In_2_S_3_ (311) in sample IPM3. Thus HRTEM results assured the creation of In_2_S_3_–MoS_2_. The information regarding the structural profile of the pristine MoS_2_ sample is represented in Fig. 2 supporting information).

**Figure 6:**
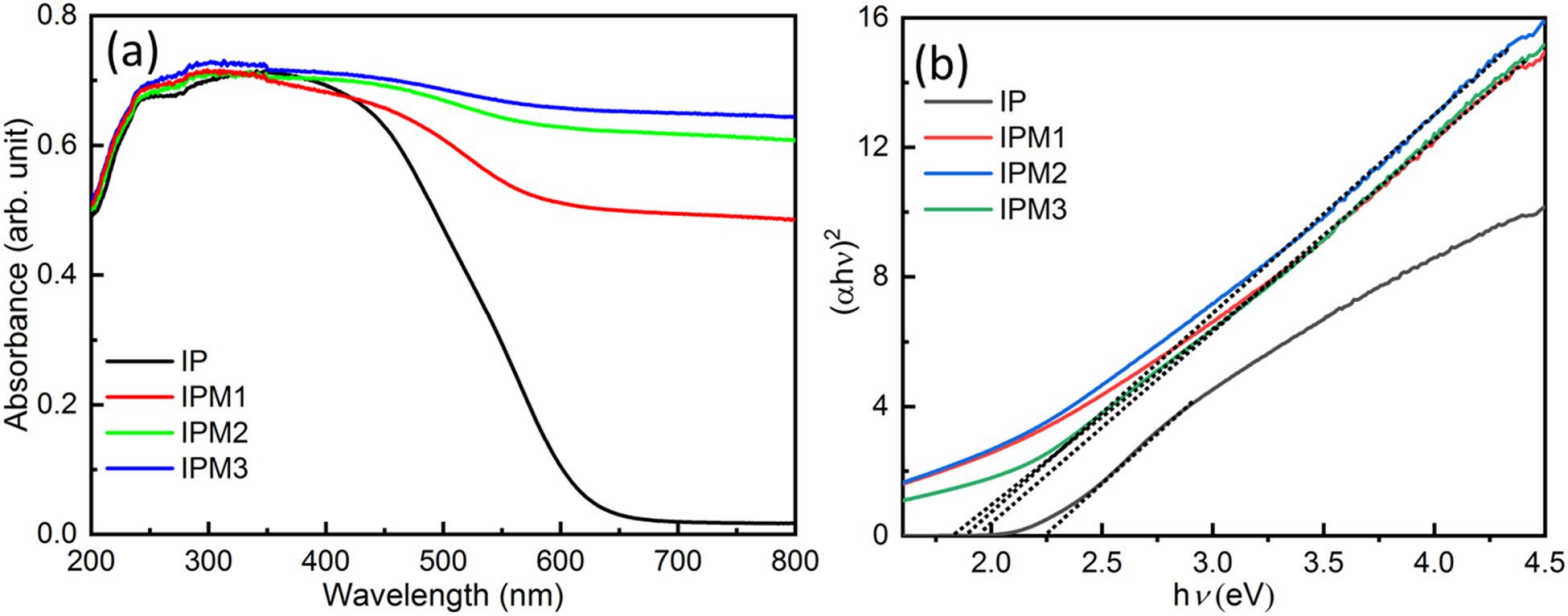
(**a**) UV-DRS studies depicting the variations in the optical absorbance profile for sample IP, IPM1, IPM2, and IPM3, (**b**) Tauc plots for sample IP, IPM1, IPM2, and IPM3.

The optical profile of pristine In_2_S_3_ nanosheets and MoS_2_ modified In_2_S_3_ is illustrated in Fig. 6a. The optical absorption curve of sample IP reveals a broad peak form 250–600 nm. With the modification of MoS_2_ in In_2_S_3,_ the optical absorption enhanced significantly (sample IPM1). For sample IPM2, the optical absorption is further increased and found higher than IP and IPM1. The optical absorption spectrum for sample IPM3 indicates the highest absorption compared to sample IP, IPM1, and IPM2. Optical absorption studies manifest a remarkable improvement in MoS_2_–In_2_S_3_ nanohybrids as compared to pristine In_2_S_3_. To explore the bandgap engineering in MoS_2_–In_2_S_3_ nanohybrids Tauc plots, studies were carried out and presented in Fig. 6b. Tauc plots suggest the narrowing in the bandgap in MoS_2_–In_2_S_3_ nanohybrids as compared to pristine In_2_S_3_. The computed bandgap for sample IP, IPM1, IPM2 and IPM3 are 2.25 eV, 1.94 eV, 1.89 eV and 1.82 eV, respectively.

**Figure 7:**
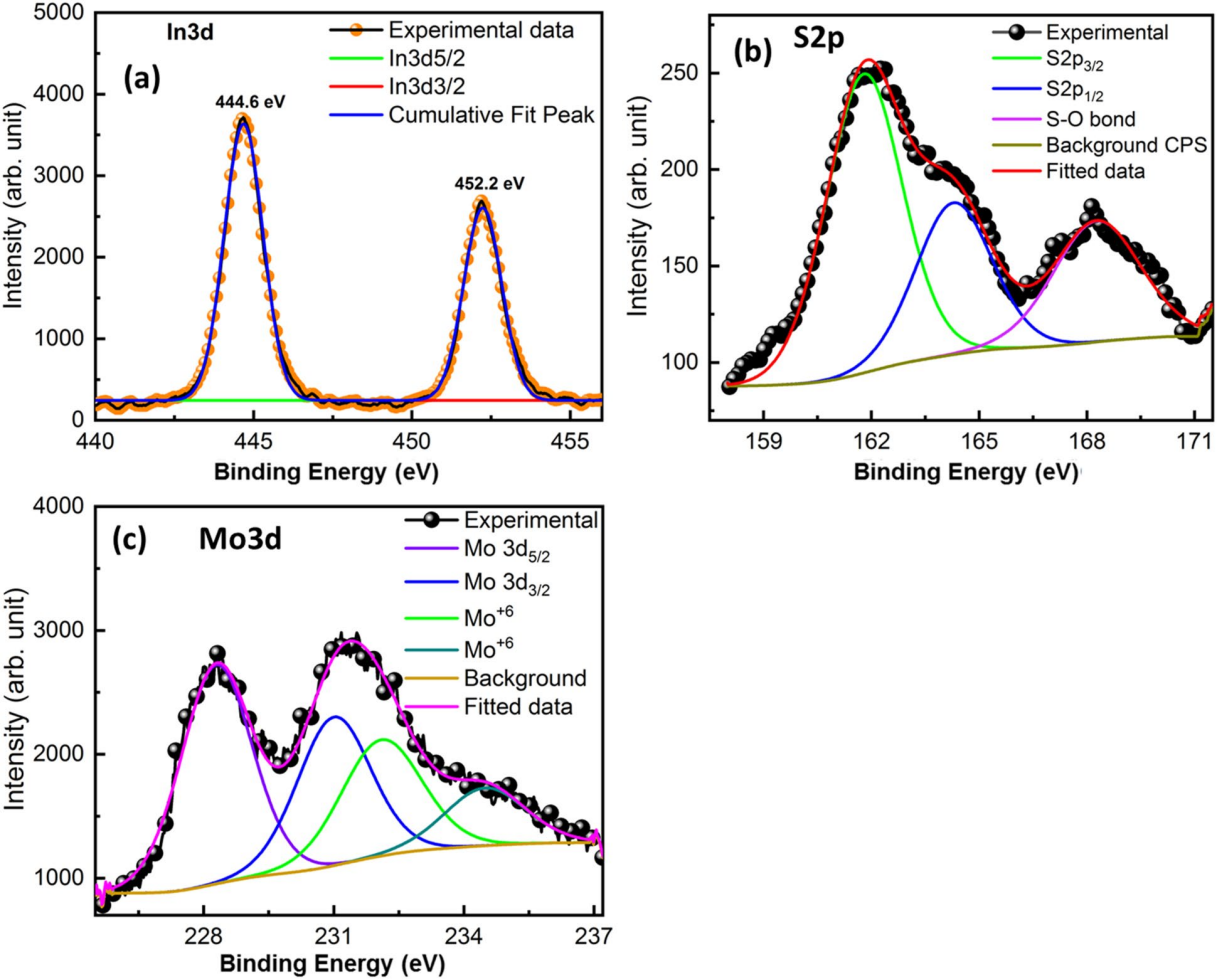
(**a**) Fitted XPS spectrum of In3d, (**b**) Fitted XPS spectrum of S2s, (**c**) Gaussian fitted XPS of Mo3d.

The valance state and surface chemical composition of MoS_2_–In_2_S_3_ nanohybrids were determined through the XPS and illustrated in Fig. 7. Gaussian fitted XPS spectra for In3d, S2p and Mo3d were presented in Fig. 7a–c, respectively. Figure 7a reveals that the XPS spectrum for In3d consists of two distinct peaks at 444.6 eV and 452.2 eV, which can be assigned to the In3d_5/2_ and In3d_5/2,_ respectively^44^. Gaussian fitted S2s spectrum reveals the three peaks at 161.8 eV, 164.3 eV and 168.2 eV (Fig. 7b). The peaks at 161.8 eV and 164.3 eV can be attributed to the S2P_3/2_ and S2P_1/2_ sequentially and affirms the presence of the S2p state of the sulfur atom^38,39^. Apart from this, a broad peak at 168.2 eV assured the existence of the S–O bond^45^. This bond inferred the partial oxidation of sulfur in the hydrothermal process^45–47^. The fitted spectrum of Mo3d shows the distinct four peaks at 228.3 eV, 231.0 eV, 232.1 eV, and 234.5 eV (Fig. 7c). The major peaks at 228.4 eV and 231.3 eV corresponds to the Mo3d_5/2_ and Mo3d_3/2,_ respectively^41^. The peaks at 232.1 eV and 234.5 eV can be attributed to the existence of Mo^+6^ states in the Mo3d spectrum^46,48^. Both peaks arise due to the incomplete reduction of Mo precursors in hydrothermal reaction^48^. Zou et al.^48^ reported the formation of MoS_2_/RGO nanocomposites through the hydrothermal method. In their XPS study, they have also observed the Mo^+6^ peaks due to incomplete reduction of Mo precursors. Moreover, it is possible that edges of the MoS_2_ or defect state are bonded with oxygen and give rise to the peaks corresponds to the Mo^+6^ states^49^. XPS studies indicate the presence of Mo, In, and S in the state of Mo^+4^, In^+3^, and S^-2^ state in the MoS_2_–In_2_S_3_ nanohybrids, respectively. XPS results explicitly suggest that no shift takes place in the recombination process of MoS_2_–In_2_S_3_ nanohybrids.

**Figure 8:**
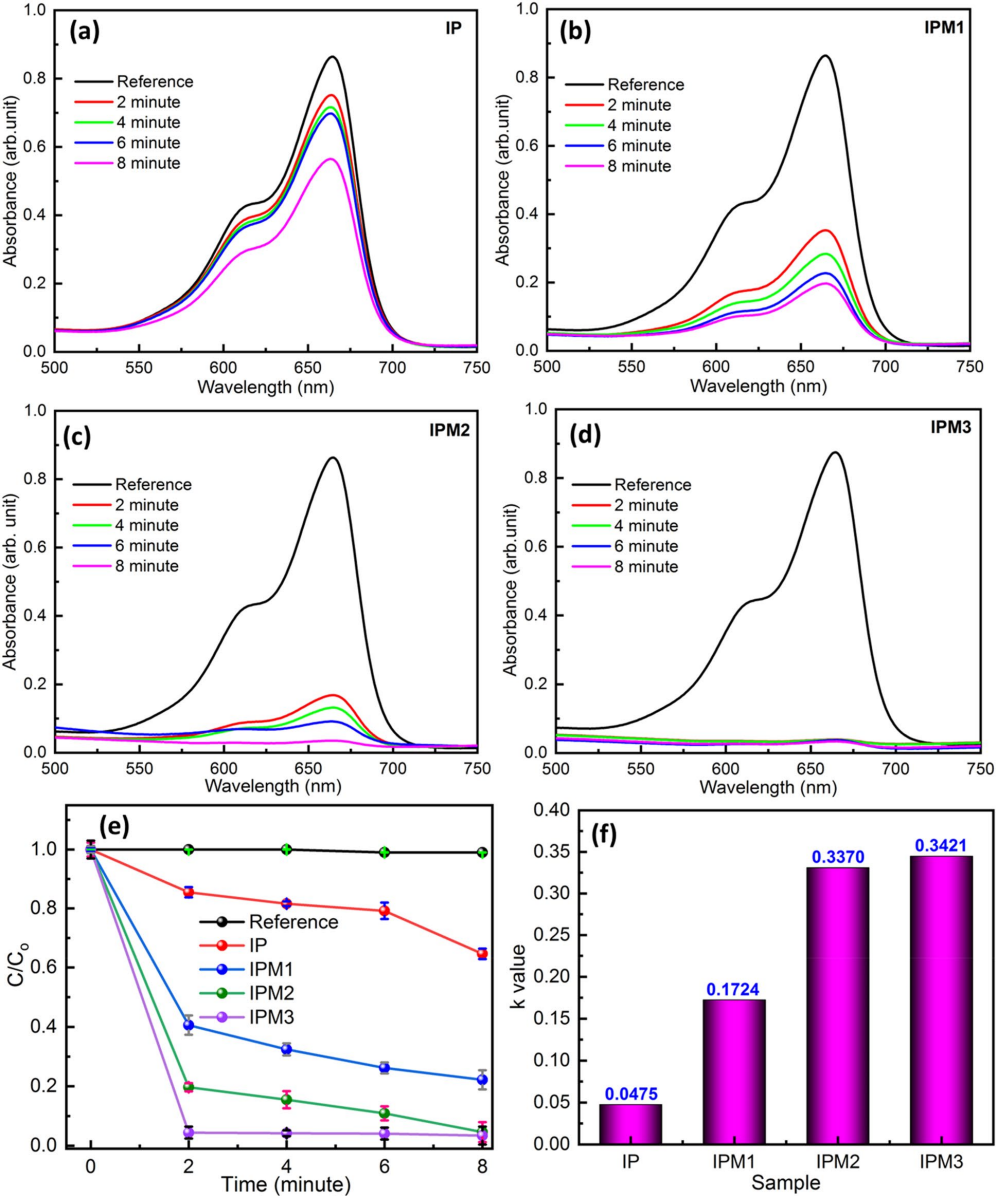
(**a**–**d**) Optical absorption spectra of MB molecule by employing different photocatalyst sample IP, IPM1, IPM2, and IPM3, (**e**) Photodegradation rate kinetics of MB molecule degradation by employing sample IP, IPM1, IPM2, and IPM3, (**f**) Rate constant (k) values of the breakdown of MB molecule solution using sample IP, IPM1, IPM2, and IPM3.

**Figure 9:**
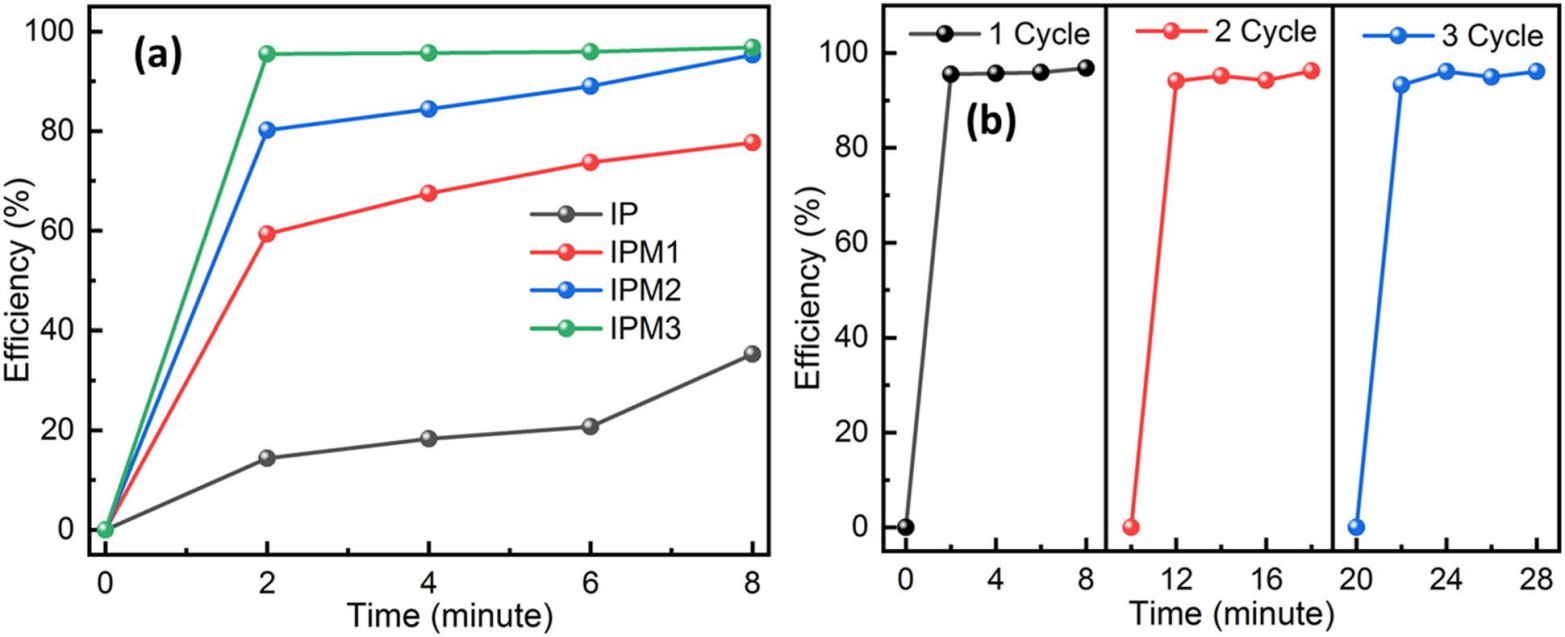
(**a**, **b**) Photodegradation efficiency of sample IP, IPM1, IPM2, and IPM3 under sunlight, (**b**) Reusable photocatalyst test for sample IPM3 indicating its stability.

**Figure 10:**
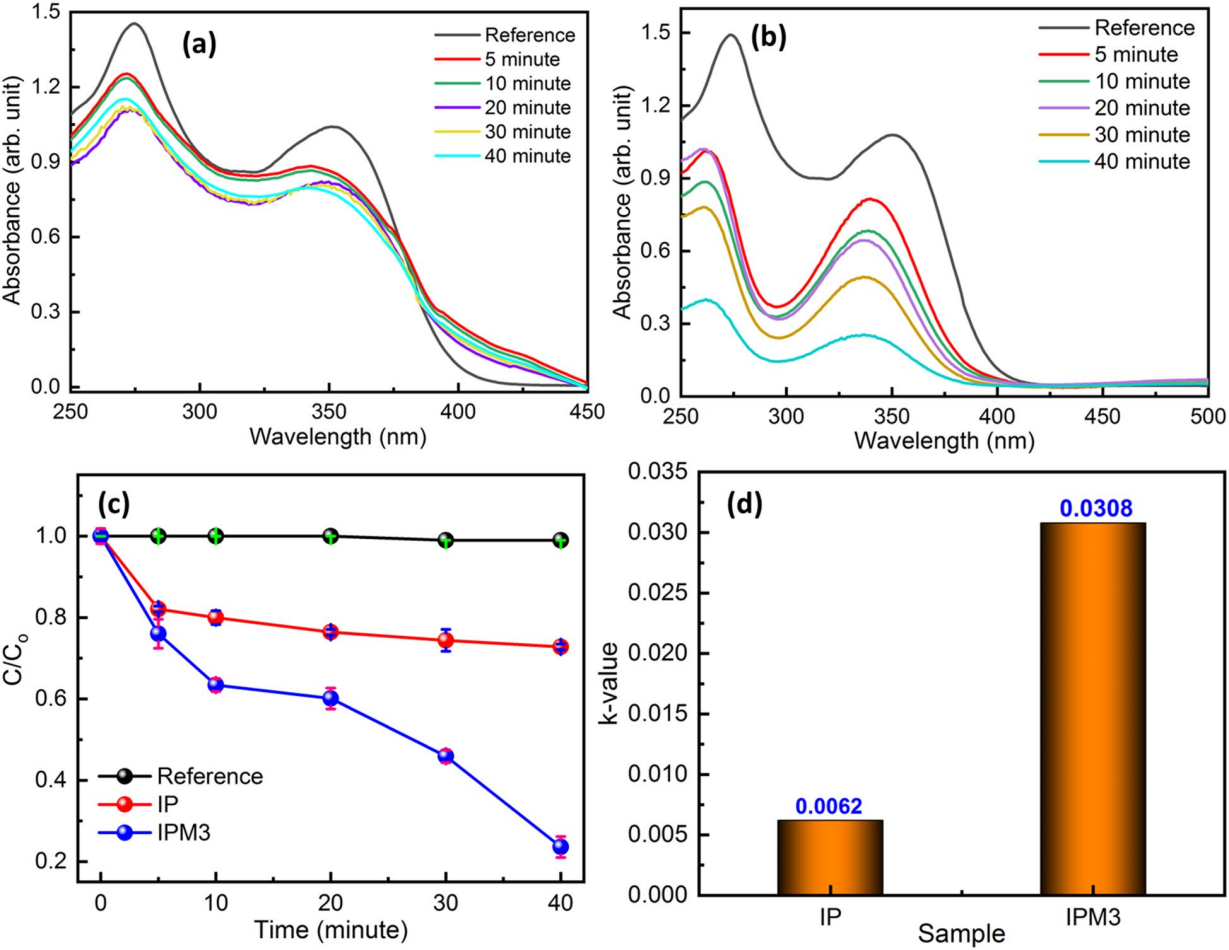
(**a**, **b**) Optical absorption spectra of OTC-HCl molecule by embracing IP and IPM3 photocatalyst respectively. (**c**) Degradation rate kinetics of OTC-HCl molecule using sample IP and IPM3, (**d**) Bar graph revealing the rate constant value for the breakdown of OTC-HCl molecule under sunlight using photocatalyst IP and IPM3.

We investigated the sunlight-induced photodegradation ability of the In_2_S_3_ nanosheets and MoS_2_ modified In_2_S_3_ samples by the degradation of MB molecule solution. Figure 8a–d depicts the optical absorption spectrum of MB molecule solution employing sample IP, IPM1, IPM2, and IPM3. A UV–visible absorption study implies that sample IPM3 exhibits the highest photocatalytic efficiency compared to sample IP, IPM1, and IPM2. Sample IPM3 is found 96.8% efficient for the breakdown of 10 µM MB molecule solution in 8 min, while sample IP, IPM1, and IPM2 are found capable of decomposing 35.3%, 77.7%, and 95.3% of 10 µM MB molecule solution in 8 min (Fig. 9a). Figure 8e shows the modulations among the photodegradation kinetics of sample IP, IPM1, IPM2, and IPM3. The photodecomposition capability of pristine MoS_2_ nanoflowers for MB pollutant molecules was measured and presented in (Fig. 3a supporting information). The calculated photodegradation efficiency of pristine MoS_2_ is 62.2%. Photodegradation rate kinetics reveals the higher photodegradation performance of sample IPM3 as compared to other photocatalyst samples. In order to found out the rate constant value of each photocatalyst sample, linear fitting of logarithm value of C/Co as a function of exposure time were plotted. The rate constant value for sample IP, IPM1, IPM2, and IPM3 is depicted in Fig. 8f. The calculated k values for sample IP, IPM1, IPM2, and IPM3 are 0.0475/ min, 0.1724/min, 0.357/min, and 0.3421/min, sequentially while the k value for pristine MoS_2_ nanoflowers is found to be 0.1673/min (Fig. 3b supporting information). Photocatalytic studies affirm that sample IPM3 attain 2.70 times better photodecomposition performance as compared to pristine sample IP. The rate constant value for sample IPM3 is 7.2 times and 2.04 times the k value of sample IP and pristine MoS_2_. Photodecomposition results assure the improved degradation nature of the IPM3 sample as compared to other prepared photocatalyst samples. The stability of the most efficient synthesized sample (IPM3) was explored through the usage of sample IPM3 for three cycles of the photocatalytic reaction process. After three runs of photodegradation reaction, the efficiency of sample IPM3 is constant, which indicates their stable nature (Fig. 9b). To reveal the high photodegradation capability of the most efficient sample IPM3, the photodecomposition of OTC-HCl molecules was explored. Figure 10a,b indicates the optical absorption results of photodegradation results of OTC-HCl molecule solution using sample IP and IPM3, respectively. Photodegradation rate kinetics assures the high photodecomposition performance of sample IPM3 as compare to sample IP. It has been computed that sample IP can decompose the 27.1% efficient while sample IPM3 shows the decomposition of 76.3% of 0.3 mg/mL of OTC-HCl solution in sunlight (Fig. 10c). The k-values for sample IP and IPM3 are 0.00621/min and 0.0308/min sequentially (Fig. 10d).

**Figure 11:**
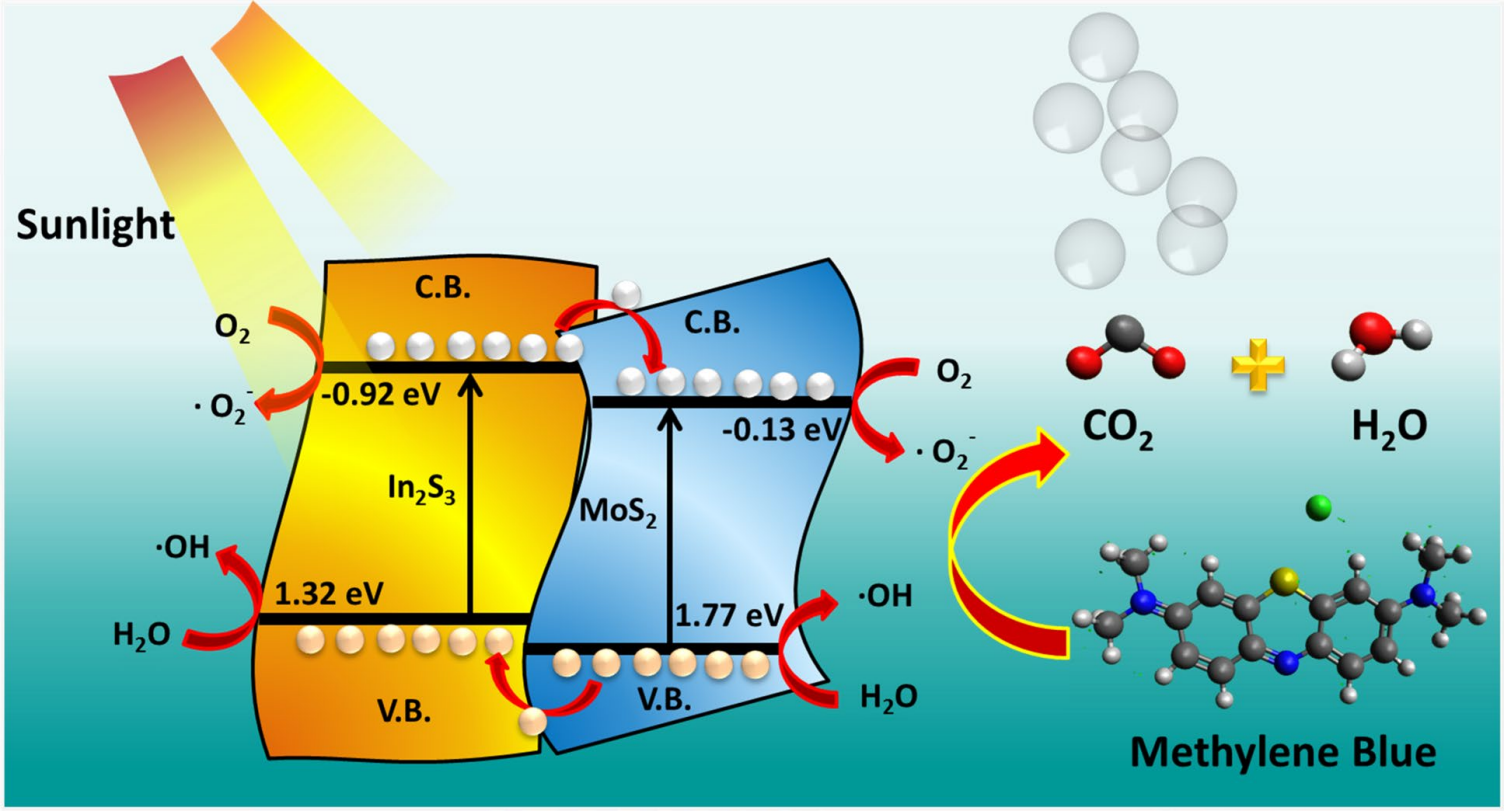
Energy level schematic diagram of In_2_S_3_–MoS_2_ nanohybrids depicting the synergistic effect among In_2_S_3_ nanosheets and MoS_2_ nanoflowers under sunlight.

To determine the charge transfer profile in In_2_S_3_–MoS_2_ nanohybrids during the photocatalytic reaction, a schematic diagram has been presented (Fig. 11). Under sunlight irradiation, In_2_S_3_ sheets and MoS_2_ flakes are activated and generate the electron–hole pair in their respective conduction and valence bands. In the next step, the photoinduced electron in the conduction band of In_2_S_3_ moves towards the conduction band of MoS_2_ due to the band alignment positions, which in turn maintain the synergistic effect and control the recombination rate in In_2_S_3_ nanosheets. The VB and CB positions for In_2_S_3_ nanosheets and MoS_2_ nanoflakes are obtained through the following relation (1).

1 $$ E_{CB} = \chi - E^{e} - 0.5E_{g} $$

where E_g_ indicates the bandgap value of the 2D layered materials (In_2_S_3_ and MoS_2_) while E^e^ stands for the energy on the hydrogen scale (4.5 eV), and χ shows the electronegativity of the 2D layered nanostructures (In_2_S_3_ ~ 4.7 eV and MoS_2_ ~ 5.32 eV). The computed VB and CB values for In_2_S_3_ nanosheets are 1.32 eV and − 0.92 eV, sequentially, while CB and VB values for MoS_2_ nanoflakes are − 0.13 eV and 1.77 eV, sequentially.

Apart from the efficient charge separation process, the density of electrons in CB of MoS_2_ enhanced remarkably; consequently, the formation rate of superoxide radicals increased. The high concentration of superoxide radicals primarily affects the photocatalytic reaction and accelerates it significantly. Similarly, due to the synergistic effect among In_2_S_3_ and MoS_2,_ the enhanced production of holes interacted with the water molecule and created the hydroxyl radicals with high concentrations. The high density of hydroxyl radicals interacts with the MB molecule and degrades it. Thus the synergistic effect in In_2_S_3_ sheets—MoS_2_ flakes control the charge separation and improve the photodegradation ability. To recognize the active species responsible for the improved photocatalytic activity of In_2_S_3_-MoS_2_ nanohybrids, charge trapping studies were embraced and presented in Fig. 4 (supporting information) Scavenger studies inferred that BQ and CN largely quench the rate of photocatalytic reaction kinetics of sample IPM3. Thus it can be concluded that superoxide and electrons majorly influence the photocatalytic reaction kinetics. In order to further determine the stability of sample IPM3 during the reusable photodegradation process, their structural characterization was explored and depicted in Fig. 5 (supporting information). Raman studies and TEM images assure the high stability of sample IPM3.

In the present report, an In_2_S_3_–MoS_2_ nanohybrids with an effective charge separation effect is successfully fabricated. The density of MoS_2_ nanostructures over In_2_S_3_ nanosheets was precisely varied and used for the decomposition of MB and OTC molecules under solar light illumination.

The highest bandgap narrowing occurs in sample IPM3, which also indicates the efficient charge separation. Raman and XRD studies also reveal that the density of MoS_2_ over In_2_S_3_ nanosheets was increased from sample IPM1 to IPM3. For sample IPM3, high numbers of heterojunction of MoS_2_ and In_2_S_3_ are responsible for the extremely high sunlight-induced photodegradation capability towards MB molecules solution. Thus sample IPM3 decomposes 96.8% 10 µM of MB and 76.3% of 0.3 gm/mL OTC molecules solution in 8 min and 40 min sequentially under solar light illumination. Liu et al.^33^ reported the formation of MoS_2_ nanodots functionalized In_2_S_3_ nanoplates and used them for the photoelectrochemical application. They have found that MoS_2_/In_2_S_3_ heterojunction with effective charge exhibited better performance as compared to pristine MoS_2_ and In_2_S_3_. Wang et al.^34^ synthesized MoS_2_ functionalized In_2_S_3_ nanostructures for the Cr^+6^ removal using a photocatalysis process. They have demonstrated that MoS_2_ functionalized In_2_S_3_ nanostructures exhibit 3.2 times better photocatalytic activity than pristine In_2_S_3_.

## Conclusions

We have successfully engineered In_2_S_3_–MoS_2_ nanohybrids using a simple hydrothermal method. The density of MoS_2_ flakes was tuned over the surface of In_2_S_3_ nanosheets. Improvement in the optical absorption and significant bandgap narrowing tremendously contribute to improving the charge separation in 2D-layered In_2_S_3_–MoS_2_ nanohybrids. Optimized In_2_S_3_–MoS_2_ nanohybrids reveal the outstanding sunlight-driven photodecomposition performance for MB and OTC pollutant molecules solution. In_2_S_3_ nanosheets combined with the MoS_2_ nanoflakes decompose the 96.8% 10 µM of MB and OTC (0.3 mg/mL) solution in 8 min and 40 min, respectively. The photodecomposition capability of 2D-layered In_2_S_3_–MoS_2_ nanohybrids was found 2.7 times higher as compared to pristine In_2_S_3_ nanosheets. Charge trapping studies inferred that superoxide radicals and electrons density majorly take part to improve the photodegradation ability of In_2_S_3_–MoS_2_ nanohybrids. These results are very significant and unique, which demonstrated the outstanding photocatalytic activity of 2D layered In_2_S_3_–MoS_2_ nanohybrids towards chemical and pharmaceutical waste.

## Supplementary Information

Supplementary Information.
