# Supplementary material for: Enhanced sunlight driven photocatalytic activity of In2S3 nanosheets functionalized MoS2 nanoflowers heterostructures
Source: Sci Rep. 2021 Jul 28;11:15352. doi: 10.1038/s41598-021-94966-z (PMC8319311; doi:10.1038/s41598-021-94966-z)
Supplement: Supplementary file 1 — Supplementary Information. [file 41598_2021_94966_MOESM1_ESM.docx]

**Supporting Information**

**Figure 1**


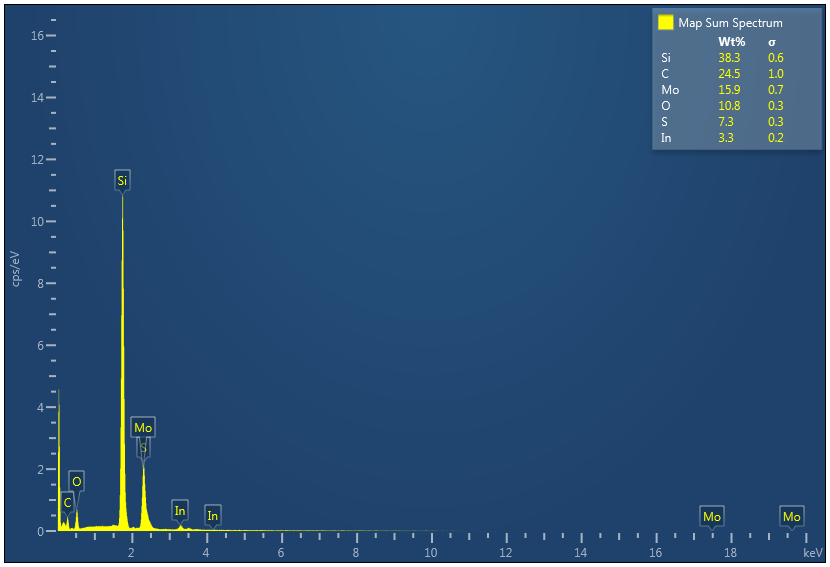


**Figure 1:** EDS spectrum for sample IPM3 indicates the percentage of each element and revealing the formation of MoS_2_/In_2_S_3_ nanohybrids.

**Figure 2**

**
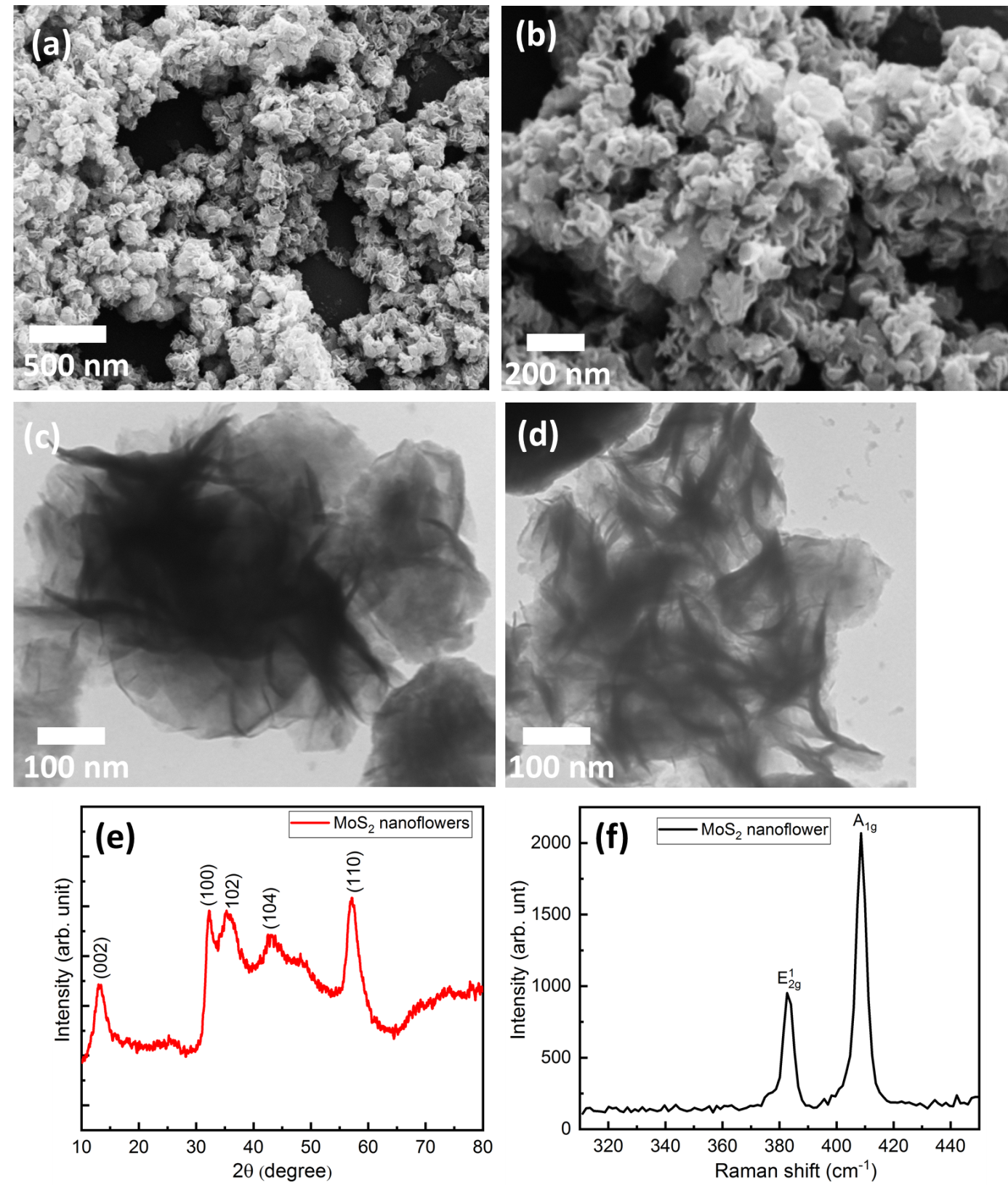
**

**Figure 2:** (a-b) SEM micrographs of pristine MoS_2_ nanoflowers sample, (c-d) TEM images of pristine MoS_2_ nanoflowers revealing assembly of nanoflakes, (e) XRD result of pristine MoS_2_ sample, (f) Raman spectrum of pure MoS_2_ sample.

**Figure 3**

**
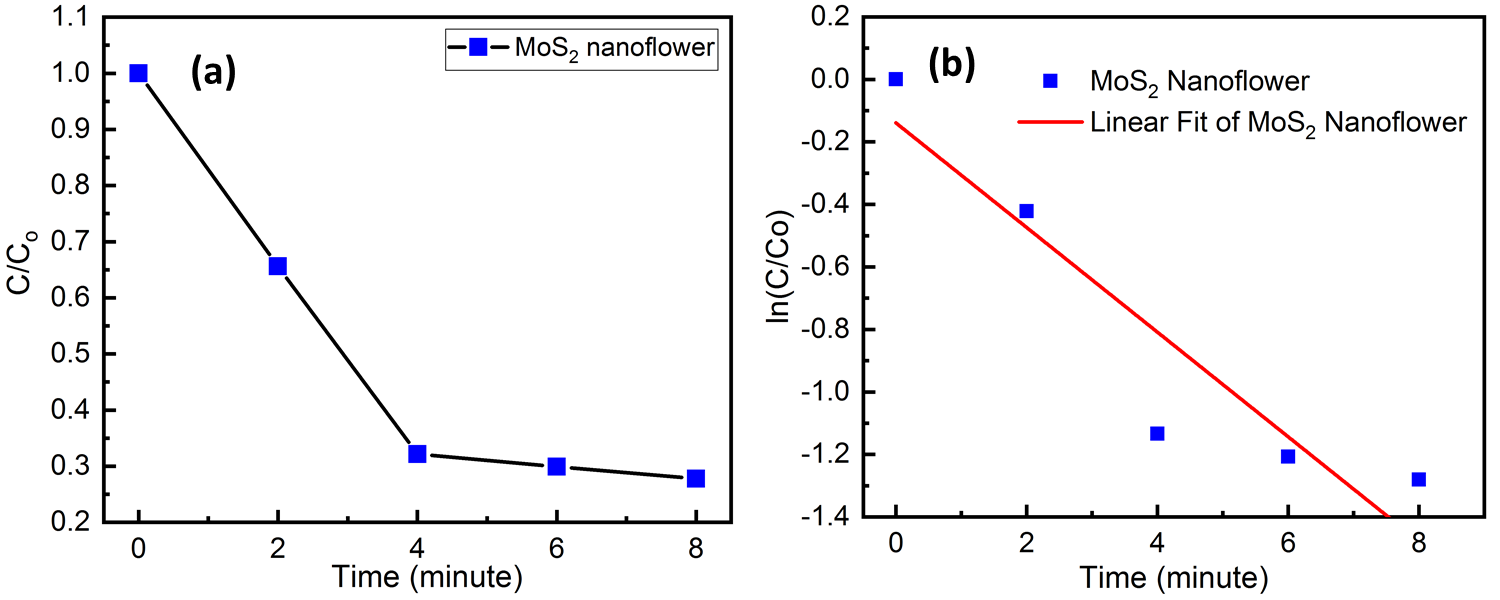
**

**Figure 3:** Photodegradation rate kinetics curve for MB pollutant molecule solution using pristine MoS_2_ nanoflowers under sunlight exposure (b) Curve among the ln(C/C_o_) and illumination time indicating the rate constant value (k).

**Figure 4**


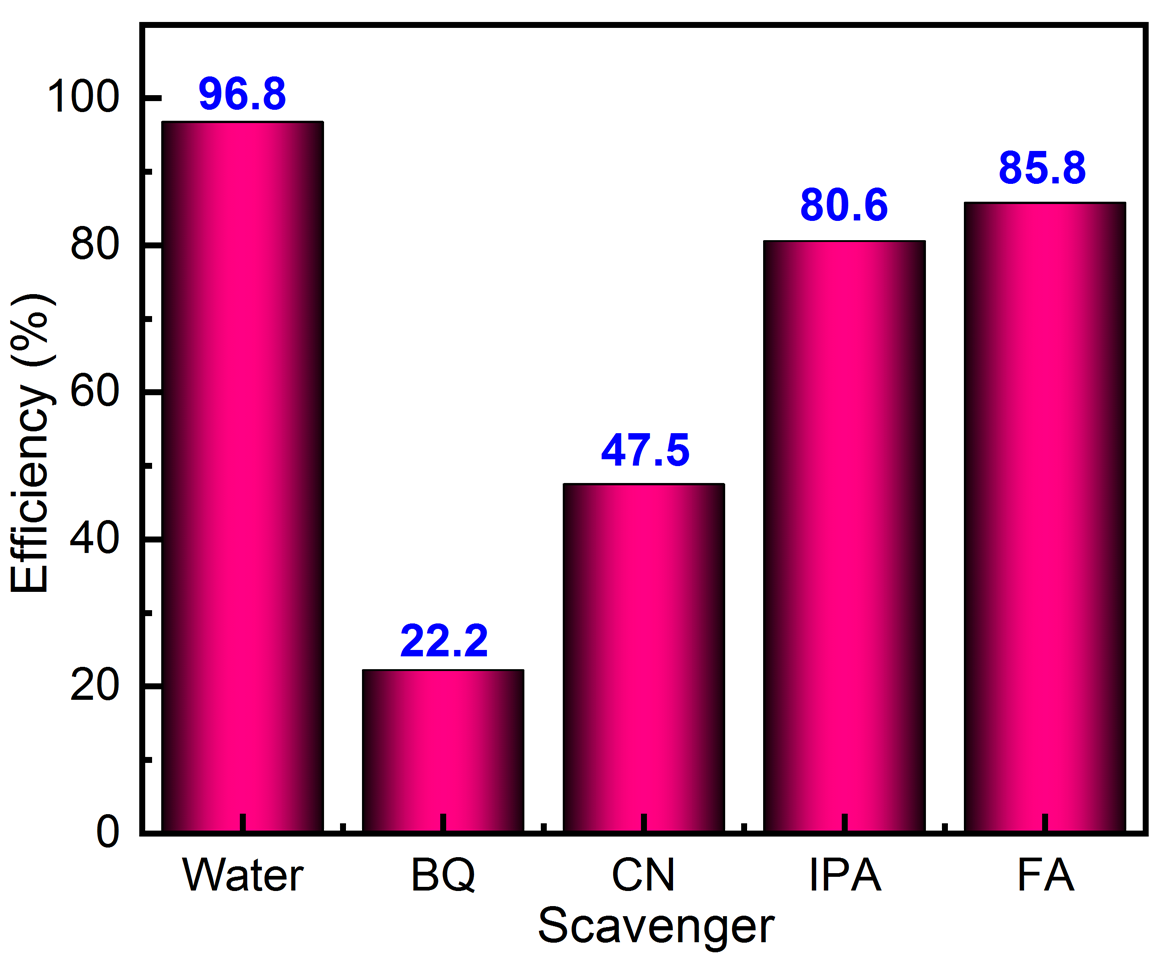


**Figure 4:** Bar graph indicating the photodegradation efficiency of IPM3 sample towards MB molecules solution with different scavengers (benzoquinone (BQ), copper nitrate (CN), formic acid (FA), and Isopropanol alcohol (IPA)).

**Figure 5**

**
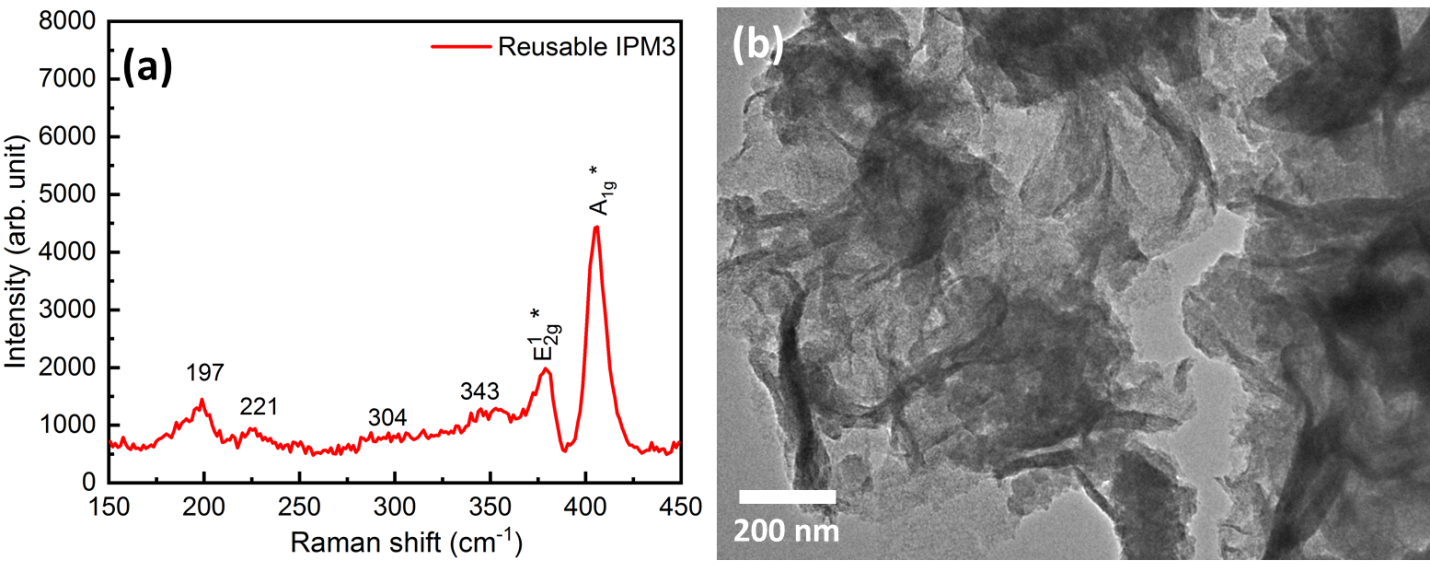
**

**Figure 5:** (a) Raman spectrum of In_2_S_3_-MoS_2_ nanohybrids after reusable photocatalytic test, (b) TEM image of In_2_S_3_-MoS_2_ nanohybrids after the three runs of photocatalytic reaction.
